# The Determinants of Translational Medicine Success - A Managerial Contribution

**Published:** 2013-05-06

**Authors:** Francesco Polese, M. Capunzo

**Affiliations:** 1 Dept. of Business Studies (Management & Information Technology), University of Salerno, Italy - ( fpolese@unisa.it ); 2 Dept. of Medicine and Surgery, University of Salerno, Italy ( mcapunzo@unisa.it )

**Keywords:** Translational medicine, health networks, service co-creation, innovation management, systems thinking

## Abstract

Scope of Translational Medicine is to speed the development of new compounds of medical protocols and/or treatments to improve patient’s quality of life. Translational medicine represents the synergy between epidemiology, basic research and clinical trials, and is based upon Innovation Management and Research Development in medicine. Being the speed and progression up to the patient a key issue of Translational Medicine, the innovation process ought to be pursued according to rigorous protocols embedded on a research development path capable of decreasing the lead time at the most. Translational Medicine represents a goal to be pursued by all involved actors, from academic researchers to clinicians, patients and others than can be seen as a network of co-creating actors engaged for the ultimate patient benefit. To underpin Translational Medicine advantages and determinants, the paper approaches the issue by adopting a systems thinking perspective, capable of highlighting the key issues to be considered.

## I. SCIENTIFIC AND CLINICAL GOALS OF TRANSLATIONAL MEDICINE

There is growing evidence of the importance of translational medicine in the improvement of patient outcome [ 1 , 2 ] . Reducing human disease and mortality is, in fact, the end purpose which translational medicine is generally and commonly recognized to be oriented to [ 3 , 4 ] .

However, the patient outcome perspective ought not to be the only interpretation key of translational medicine, since it produces different values for different actors looking at various aspects of this medical approach [ 5 ] . For academics, it represents the chance to confirm and validate novel concepts or to find new ones out with the hope they could turn into effective clinical applications and be relevant to human disease [ 6 ] ; for patients as well as for clinicians, it refers to the need of accelerating the capture of the biomedical research benefit, wishing the gap between “what we know and what we practice” to be bridged [ 7 , 5 ] ; for those who invested in, translational medicine provides financial returns [ 8 ] .

Despite the variegated list of benefits and stakeholders, it seems possible to identify a unifying purpose, capable of complying with the expectations and needs of all involved actors [ 5 ] once we higher the level of observation and analyze its beneficial effects on society. The ultimate goal of translational medicine, in fact, may be identified in the development of new treatments and insights for the improvement of health across populations [ 9 , 10 , 11 ] . This implies that translational medicine (also called translational research) not only aims to produce values and bring them to the patient. Its essence lies in validating the potentiality of novel discoveries whereas enhancing the success, feasibility and efficiency of discovery validation. In other words its ultimate goal lays in identifying in the process of clinical testing to human disease (through direct observation) what the obstacles are [ 12 , 13 ] and allowing basic scientists as well as physicians to share their expertise to indentify and compare the challenges at the interface between basic and clinical investigation, proposing integrated and integrating solutions to increase the efficiency of the process [ 8 ] .

This means that the scientific phase of the research and the applied one both equally contribute to reach the common purpose of translational medicine - which is claimed to be finding alleviation to human suffering [ 6 ] . As confirmed by Littman [ 5 ] , “translational research should be seen as enabled by ongoing efforts in basic and clinical research and not competing with them”. Translational medicine draws results about disease by clinically testing the viability of novel hypothesis [ 5 , 6 ] . Such hypothesis may reveal to be wrong or irrelevant to the care purposes. Currently, the problem is that if “in times of abundance, efficiency may not be the highest priority, and scientists might have the chance to indulge the luxury of speculative adventures in the world of the unknown [...] in these times of restricted funding opportunity, it behooves us to select our scientific challenges parsimoniously by constantly confronting our intuitions with the reality of human pathology” [ 6 ] . In other words, application criteria must ensure positive results in a framework of appropriateness, financial sustainability, interventions equity and integration.

Hence translational medicine success encompasses not only scientific and operational, but also financial, ethical, social, regulatory and legislative contingencies [ 5 ] .

## II. INNOVATION MANAGEMENT AND TECHNOLOGY TRANSFER IN HEALTH

Medicine, and health treatment advance lays upon innovation. Research and development programs involve most of health organizations in most of countries, and are expensive and long lasting, due to results uncertainty of many research pathways aimed to mitigate the negative effects of important diseases affecting human population.

Innovation in health contexts results from both scientific and technological progress and often is strictly depending on their reciprocal inferences. In fact, health innovation is the result of both biomedical research (genomics, neuroscience, molecular oncology, etc..) and technology (medical diagnostics, biotechnology, health informatics, electronic devices, etc..). Consider, e.g., the following innovation advances in health fields:

- proteomic;
- biomolecular-diagnostic;
- pharmacogenetics;
- diagnostic imaging [ 14 ] .

Technological developments in these mentioned fields are able to characterize and deeply transform the results of medicine as well as the processes of care. At the same time innovation and technology transfer determine strong implications on health services and consequently managerial, organizational [ 15 ] and operational needs of modern health systems, with a relevant impact on both health and costs. Managing health care organizations is mainly about managing innovation [ 16 , 17 ] . Therefore, innovation and managerial dimension of health services are highly interconnected factors. In this mainframe we may observe how the issue of sustainability of an health system calls for efficient government models and practices. In other words, the sustainability of the health systems largely depends on the ability to govern the entry as well as the implementation and results of innovative technologies both in clinical practice and in science.

Hence, although exploiting existing competencies may provide short-term success, competence exploration may become the hindrance for organization’s long-term viability and competitive advantage [ 18 ] . Innovation, in fact, can bring substantial benefits to both economic variables (although in the long-run) and qualitative ones, such as in the training of professionals, e.g. projects of lifelong learning, aimed at ensuring a high level of end quality performance.

Thus, effectiveness and efficiency of the new technologies are guaranteed by technology transfer. However technology is something complex to transfer, even more in healthcare as a turbolent, challenging environment. In this context, in fact, solutions should be scalable, replicable and versatile to different operative scenarios and users. Moreover health advances are constrained by financial issues (as rising costs and lack of funds), limited access to information, coordination problems. With reference to this last aspect, it is possible to state that processes, components and even people of a health system are not always coordinated, and errors in duplications, missing information or wrong one occur. Since many players are involved in healthcare innovation processes, each one with different priorities and finalities, it is necessary to overcome such divergences that before being operational, refer to language, education, culture, purposes. Currently, it is technology transfer and integration to make the healthcare systems smarter [ 19 ] – that is – with better, faster and more detailed information within the actors involved in, reducing errors and inefficiencies in the transfer, allowing the system to capture, manage and turn data into relevant information in real time. Thanks to ICT (Information and Communication Technology) platforms, including people, processes and knowledge, alignment of scope is created as well as reduction of coordination and transaction costs between involved actors [ 20 ] . Consider, in this regard, the strong advances accomplished by *eHealth* (or health in net) techniques. *eHealth* is the practice of healthcare through the support of informatics tools, highly skilled professionals and practitioners-patients communication techniques. That represents a major challenge in the field of technology transfer. *eHealth* services are in fact aimed at:

- improving the efficiency of primary health care through the integration in the network of health professionals;
- supporting the integration of various health organizations at a local level in order to facilitate the processes of care;
- facilitating access to services by users, enhancing and facilitating the choice of the citizens through the interoperability between the systems;
- supporting the control of health expenditure, by monitoring the demand for health services.

Hence, *eHealth* , and in general ICT, can be considered as a tool for health professionals to improve not only the quality of care but also the production efficiency of the health sector [ 21 ] , with positive effects in terms of sustainability of health systems in their entirety.

Thus, again, all stakeholders gain from technology advances, implementation and transfer. Definitively, e-based technologies favor the generation of high quantity data representing data gathering, registration, elaboration and dissemination models [ 22 , 23 ] . Results seem to be encouraging but the challenge even concerns the ability to exploit such potentialities. At organizational level, it implies the creation of a collaboration and relational culture. Innovation networks can be referred to as inter-organizational networks that ought to be managed appropriately in order to bring results [ 24 ] .

## III. HEALTH SERVICE EMBEDDED IN NETWORKS OF CO-CREATING ACTORS

Health is a machine producing and consuming performance (services) to create care [ 25 ] . However we may decline care in various specific results, and health systems, shaped either on the model of privatized social security (of U.S. origin) or universal model (European origin), offer services including:

- disease prevention [ 26 ] ;
- food safety;
- primary care and continuity of care;
- emergency and urgency;
- hospital care;
- rehabilitation;
- pharmaceutical care [ 27 , 28 ] ;
- care for the elderly;
- other (blood transfusion services, mental health care, palliative care, vegetative states, drug addiction and alcoholism).

Delivery of such services necessarily requires the active participation of various stakeholders, including institutional actors (local health authorities, hospitals, districts, nursing homes, municipalities, volunteer associations) which are responsible for the care and the provision of services; other national and local agencies of planning and control (Region, State, local entities) which collaborate in the support and delivery of services; actors who are currently in charge of the medical and scientific training (Public Administrations, professional associations, scientific societies, trade unions of category and Universities), citizens, providers of goods and services of health organizations; others.

Each mentioned actor participates in health creation and dissemination by exchanging resources and information. Hospitals exchange with government agencies information to monitor and audit reporting activities, with its suppliers for the purchase of goods and services. Ministries and Government Agencies collaborate for the provision of benefits, facilities and incentives, or even hospitals and professionals exchange personal data files.

Researchers and diagnostics exchange programs, research and experimental studies; they share expertise, skills and know-how at the time of interface between the scientific and applied research. Accordingly, within these health service networks of actors, we may think of translational research as the bridge between academic science and clinical practice. Such a bidirectional path “from bench to bedside” and back [ 29 , 6 ] , advocates for robust, bidirectional information flow [ 30 ] and more effective collaboration involving academia, industries and patients as well [ 31 , 5 , 32 ] .

As previously mentioned, healthcare systems ultimately produce a service ^1^ : health. The work all actors do, aimed to affirm a collective orientation as the recognition of health as a public value, necessarily requires the involvement and awareness of such heterogeneity of actors as health system stakeholders. Involved and engaged actors contribute, by sharing their resources ^2^ , to the creation of public health through the sharing of goals and pathways, transforming the paradigm of clinicians and doctors from passive recipients of patients’ needs to pro-active actors engaging with patients for their benefit [ 34 ] . It is an emerging cultural approach aimed at the active promotion of health ^3^ . In innovative health context, it seems essential to build a common sense of medicine, which allows all individuals to take control of issues with relevant implications and consequences in their daily lives and, therefore, require extensive testing and acceptance. In other words, there is the necessity of introducing a “democratization” process in medicine, where partnership is the only alternative to perish for every stakeholder [ 36 ] .

More generally, all the actors of a health system are involved in the care process, promoting instances of improvement [ 37 ] . The effectiveness of this process is closely related to the interaction and cooperation between these parties, which seem to be related to strong coordination mechanisms at various levels such as operational, political, social, economic, ethical, legal.

Accordingly, we may posit that, in a more stringent service logic, the final value of health is co-created through shared activities [ 38 , 39 , 40 ] embedding all actors of the healthcare networks, who are thus defined as endogenous [ 41 , 42 ] to the health creation process. At the same time, engaged actors can be identified as dynamic, active resources, source of competitive advantage for health organization as well as of value and innovation [ 43 , 44 ] for the whole health system. Hence, actors of a health system are regarded as integrators of resources, or as entities which exchange resources benefiting from such exchange. As such interdependence is revealed, their collaboration for the creation of a shared value just as public health becomes inevitable. In other words, the logic of service leads to a concept of health as a service network, as heterogeneous configuration of actors, value propositions and exchange of information, resources and knowledge [ 45 ] that takes place within a dynamic network, through relationships and interactions, in order to create and sustain collective health as the end shared benefit.

Ultimately, the modern process of health services, of assistance approaches and shared nature, could be regarded as a cooperative game, aimed at the promotion, implementation and coordination of multi-actor contribution. Therefore, the way forward is that of a system in which the nations, regions, universities, businesses and individuals work together to improve the conditions necessary for viability, sustainability, effectiveness and efficiency of health value in health systems [ 46 , 47 ] .

## IV. A SYSTEM VIEW OF HEALTH SERVICE

A Service System (SS), as mentioned, results to be strongly interconnected and characterized by multi-actor interactions [ 46 ] and, thus, may be interpreted as an open system [ 48 ] , capable of improving its equilibrium through the acquisition, share and supply of resources. Hence a National Health Service observed through systems lenses in a more ample set of relationships, may be defined a System embedded in a more general one due to opening of its boundaries and the engagements with an increased number of actors/entities [ 46 ] . Among these we may identify: citizens, health private actors, doctors and so on; each actor, in this system, stabilize relations fulfilling needs and expectations [ 49 ] . Through these networks each SS actor may not only access to needed resources, but may as well release resources creating a prolific service exchange and, consequentially, stimulating a value creating system for the overall benefit of the health system.

In other words the suggested systems view highlights the role of relationships as promotes of competitiveness, viability and survival within service systems in general [ 50 ] and, of course, within health service systems. In light of a relational approach, in fact, each organization may be conceived as an active resource involved in reticular connections in a many-to-many logic [ 51 , 52 ] . Hence, according to this view, organizations are not isolated, but rather they are dependent upon existing relations. This network nature requires continuous improvement of interactions characterizing all networks knots, in order to effectively distribute the shared resources, collaborative advantages and cooperative strategies designing relationships based upon information, engagement, collaboration and trust.

Each system represents the result of common efforts displaced by its active elements; resources assignment and distribution, as well as the cooperation advantages and the relevance of alliances and cooperative strategies transform static networks, through the activation of relationships among actors, into a dynamic system, strengthening competitiveness and systems advantages [ 53 ] .

Positive interactions among each actor, hence, is supportive for the creation of effective health systems in which actors engage one with the other in synergy. In this optic the system appears to be more competitive as the qualification of relationships among actors grows. In other words better, stronger and smarter relationships create the best survival conditions for health systems.

## V. THE DETERMINANTS OF TRANSLATIONAL MEDICINE SUCCESS

Translational medicine context are, as mentioned, demanding contexts in which organizations ought to pursue continuous improvement and change and this, in systems terms, implies that health systems are open and strongly dynamic. Effectively this traits stimulate the search for homeostatic dynamics as a response to external change. As the world is becoming smarter, systems ought to become people-centric, information-driven, e-oriented, and reciprocal and collective satisfaction should encourage actors to cooperation and innovation. Health Service systems may hence be seen, adopting a systems perspective, as contexts in which co-creation takes place, where systems shape themselves into networks proposing shared and diffuse value for all involved actors. In order words to fulfill such a demanding goal a service logic should pervade each organization, favoring diffuse and reciprocal resource sharing, thus characterizing interactions among actors. According to this view, service may hence be interpreted not as a generous and cultural attitude. Indeed, service may be identified as a cultural attitude, as a logic, as the enabler and fundamental base of health systems, capable of valorizing experiences and translational medicine initiatives for all involved actors benefits [ 54 , 55 ] .

It has been highlighted how innovation and technology transfer affect health services, linking the quality of care to continuous improvement and to translational medicine prolific research contexts. It was noted that performance of these technological advances depends not only upon researchers’ ability to promote and develop wise research pathways, since the contribute of numerous other actors appears to be crucial.

Furthermore we have noted how these numerous actors involved in translational medicine success, appear to be interconnected in value co-creation networks, in which value and service for the patient (and the other actors) is the outcome of joint activities within the same system. In this perspective, patients, clinicians, private and public hospitals, pharmaceutical industries, institutions are source and contributors to the system’s performance. This latter, indeed, depends on the ability to establish wise and profitable relationships among each mentioned actor who, being satisfied by the system’s outcomes, easily releases the possessed resource to the system, strengthening its sustainability.

As a final consideration we observe that systems theories offer interesting insights and contributes to the understanding of value co-creation exchanges in health networks. According to systems theories, in fact, a service logic may be the enabler of harmonic interactions and satisfactory exchanges among involved actors. More efforts are needed in this directions, and we hope future research on systems theories contributes to health network understanding, and to the underpinning of translational medicine performance will pursue these challenges.

## Footnotes

1 Despite the path towards a unifying terminology does not seem finished yet a service can be defined more generally as an activity carried out by an individual or a group, that benefits others [ 33 ] . It is therefore a type of activity that provides assistance and experience for the benefit of all parties involved in a particular exchange, before, during and after it.

3 In this sense, an important contribute is attributed to *empowerment* [ 35 ] , the process of involving all the actors as active players in their path to health and wellness, participating and thereby influencing decision-making processes of care, treatment and rehabilitation.

2 Each actor possesses, and may offer, a crucial contribute by sharing various possessed resource, such as (but not limited to): knowledge, financial support, innovative solutions, effort, psychological involvement, information, professionalism, facilities, etc.
